# Evaluating the social return on investment of nature-based social prescribing to alleviate loneliness: a randomized controlled trial in Barcelona

**DOI:** 10.3389/fpubh.2026.1774155

**Published:** 2026-05-25

**Authors:** Veronika Papon, Sibylle Puntscher, Montse Masó-Aguado, Marjan Arvandi, Beate Jahn, Mat Jones, Amy Beardmore, Marc Olivella-Cirici, Laia Briones-Buixassa, Laura Coll-Planas, Jill S. Litt, Uwe Siebert, Ursula Rochau

**Affiliations:** 1 Institute of Public Health, Medical Decision Making and Health Technology Assessment, Department of Public Health, Health Services Research and Health Technology Assessment, UMIT TIROL - University for Health Sciences and Technology, Hall in Tirol, Austria; 2 Research Group On Methodology, Methods, Models and Outcomes of Health and Social Sciences (M3O), Faculty of Health Sciences and Welfare, Centre for Health and Social Care Research (CESS), University of Vic-Central University of Catalonia (UVic-UCC), Barcelona, Spain; 3 Institute for Research and Innovation in Life Sciences and Health in Central Catalonia (IRIS-CC), Vic, Spain; 4 Centre for Public Health and Wellbeing, University of the West of England, Bristol, United Kingdom; 5 General Sub-Directorate for Addictions, HIV, Sexually Transmitted Infections and Viral Hepatitis, Public Health Agency of Catalonia, Department of Health, Barcelona, Spain; 6 Research Group On Innovation in Mental Health and Social Wellbeing (ISAMBES), Faculty of Health Sciences and Welfare, Centre for Health and Social Care Research (CESS), University of Vic-Central University of Catalonia (UVic-UCC), Vic, Catalonia, Spain; 7 Institute for Global Health (ISGlobal), Barcelona Biomedical Research Park (PRBB), Barcelona, Spain; 8 CIBER Epidemiología y Salud Pública (CIBERESP), Madrid, Spain; 9 Universitat Pompeu Fabra (UPF), Barcelona, Spain; 10 Center for Health Decision Science, Departments of Epidemiology and Health Policy & Management, Harvard Chan School of Public Health, Boston, MA, United States; 11 Institute for Technology Assessment and Department of Radiology, Massachusetts General Hospital, Harvard Medical School, Boston, MA, United States

**Keywords:** Barcelona (Spain), economic evaluation, loneliness, nature-based social prescribing, RECETAS, social prescribing, social return on investment, social value

## Abstract

**Introduction:**

Loneliness is associated with adverse health outcomes, including cardiovascular diseases, depression, and premature mortality, and is unequally distributed according to social determinants. Prescribing individuals to community-based interventions in nature, often described as nature-based social prescribing (NBSP), has emerged as a promising intervention to address loneliness. This study aims to quantify the social return of investment (SROI) of the “Friends in Nature” (FiN) intervention designed to alleviate loneliness.

**Methods:**

Following key guidelines, we performed a SROI study for a NBSP intervention compared to usual care alongside the FiN trial conducted in Barcelona within the EU-H2020 RECETAS project. The SROI ratio, which measures the social value generated per monetary unit invested, and the net present value, which reflects the difference between social value and investment costs, were estimated.

**Results:**

A social return of EUR 1.48 per EUR 1 invested in the NBSP intervention was generated. The net present value was EUR 8,585 per FiN and EUR 8,496 per UC participant. The SROI ratios remained positive and above one in most sensitivity and scenario analyses, ranging from EUR 0.83 to 13.18 per 1 EUR invested.

**Discussion:**

We conducted a SROI analysis to quantify the broader benefits of investing in a NBSP intervention to reduce loneliness. This SROI ratio enables decision-makers to consider social value alongside financial value, thereby facilitating more comprehensive comparisons of public health investments.

**Clinical trial registration:**

Trial Registration: ClinicalTrials.gov, ID: NCT05488496. Registered 29 July 2022.

## 1 Introduction

Loneliness has been increasingly recognized as a major public health issue (1). It is associated with adverse health outcomes, including cardiovascular diseases, depression, and premature mortality (2). Beyond its impact on individual wellbeing, loneliness is unequally distributed across social determinants (3). Moreover, loneliness carries significant societal costs. Recent studies have highlighted both the direct and indirect economic burden of loneliness (4), with substantial health-related costs estimated in the United Kingdom (5) and in Spain (6).

Nature-based social prescribing (NBSP) has emerged as a promising intervention to address loneliness by fostering social connections and engagement with natural environments. NBSP connects individuals with non-clinical needs to community-based interventions and resources centered around nature. Activities such as hiking, bird watching, or gardening can help alleviate feelings of loneliness and promote social connection (7). Demonstrating the impact, costs, and benefits of NBSP is essential to support its integration into policy and practice, highlighting the need for comprehensive health economic evaluations (8).

The EU-funded project RECETAS has developed and tested the intervention “Friends in Nature” (FiN) as a NBSP intervention aiming to alleviate loneliness. The components of this structured intervention, also comprising elements to promote social connection and empowerment, are explained in detail in Coll-Planas et al. (9).

In addition to incorporating financial effects of an investment, a social return on investment (SROI) analysis considers social, environmental, and economic impacts to evaluate the total value of health-promoting investments. This approach acknowledges impacts on broader society. Notably, impacts that cannot be fully valued using market prices are monetized. For example, aspects such as friendship or social connectedness are assigned a proxy value to capture their contribution to overall generated social value (20).

Few studies have evaluated the SROI of social prescribing (SP) interventions (10). Two studies evaluated NBSP interventions for mental health conditions but were limited by small sample sizes and the absence of a control group (11, 12). Targeting loneliness, one study reported that a national SP intervention for people experiencing loneliness creates a positive SROI (13). Another study performed a return on investment analysis for loneliness, however, “QALYs and other health benefits were not monetised and excluded from the ROI ratio” (14). To our knowledge, no SROI study has evaluated a NBSP intervention specifically for alleviating loneliness.

Therefore, we aimed to quantify the social return generated by investment in the FiN intervention, a nature-based social prescribing intervention, in Barcelona alleviating loneliness based on empirical data from the RECETAS randomized controlled trial (RCT).

## 2 Methods

This study was conducted as part of the ‘Reimagining Environments for Connection and Engagement: Testing Actions for Social Prescribing in Natural Spaces (RECETAS)’ project (7). RECETAS is a European Union-funded project (No. 945095) aiming to evaluate NBSP interventions for people experiencing loneliness (15). The study design of the RECETAS trials has been previously published (9). Briefly, three RCTs were conducted in Barcelona (Spain), Helsinki (Finland) (16), and Prague (Czechia). The specific NBSP intervention developed and tested in RECETAS, referred to as “Friends in Nature” (FiN), included an individual interview followed by nine 2-h nature-based and group-facilitated sessions to alleviate loneliness. FiN is a holistic group-based model of social prescribing as defined in Litt et al. (17). FiN was compared with individual signposting as usual care (UC), which consisted of facilitators providing information to signpost people to nature-based activities, using the nature activities menu derived for the FiN intervention (9).

In this study, we aimed to evaluate the FiN intervention alongside the RCT conducted in Barcelona (Spain) (9). In total, 320 participants, aged 18 or older, from socio-economically deprived areas were randomized 1:1 to the FiN intervention or to UC by an independent researcher using a random allocation sequence generated by a computer (9). A diagram showing the flow of participants is presented in Appendix Figure A1 (18). Participants were required to be aged 18 years or older, experience loneliness, and be willing to participate in group activities held in nature. The intervention lasted over a time period of 10 weeks, participants were followed-up for 12 months from baseline and is evaluated retrospectively in this study. Health outcomes and costs were assessed in both the FiN and UC groups at baseline, at the end of the intervention period after 3 months, and follow-up at 6 and 12 months. Loneliness, health-related quality of life (HRQoL), and capability wellbeing were assessed. A health economic questionnaire was developed to assess healthcare resources use, in support of the planned health economic evaluations. In addition to traditional cost-effectiveness analyses, a SROI analysis is performed based on health outcomes and costs from baseline and 3 months assessment time points. SROI ratios were calculated based on the quantitative empirical data collected in the RECETAS trial in Barcelona (Spain) and supported by qualitative findings.

The SROI is expressed as the ratio of the monetized social benefits to the total costs of the intervention (19). This ratio measures how many units of monetized social value are generated for one monetary unit invested in the intervention. For example, a SROI ratio of 5:1 indicates that a social value of 5 Euros is generated by an investment of 1 Euro. We also report the net present value (NPV) of the intervention (20, 21).

This study was conducted following established principles and stages (20). Accordingly, the SROI analysis is conducted retrospectively based on the following six established stages of a SROI evaluation (20):

1 Defining the scope of the SROI analysis and identifying stakeholders.
2 Identifying and evidencing outcomes by developing a logic model showing “the relationship between inputs, outputs and outcomes” (20).
3 Assigning indicators to measure a difference in the identified outcomes. Value the differences to get a monetary proxy for the generated social value.
4 Adjusting the valued outcomes for deadweight, attribution, displacement, and drop-off to get the real impact of the intervention.
5 Calculating the social value by multiplying the adjusted outcomes by the number of people experiencing each outcome; calculating costs of the intervention; discounting the outcomes (and costs) over the duration of the intervention to get the present social value of each outcome (and costs); estimating the SROI ratio by dividing the total present social value by the total costs and the NPV by subtracting the costs from the present social value; and testing results in sensitivity analyses.
6 Reporting and publishing the SROI analysis to create impact (20).

### 2.1 Scope of the SROI analysis and identifying stakeholders

The scope of this SROI analysis was to quantify the social return generated by the trial in Barcelona (Spain) of the RECETAS project and to inform healthcare decision makers on the social return of their investment in NBSP strategies.

Experiencing a material change as a result of the NBSP intervention as a criterion for stakeholder selection leads to the identification of the primary stakeholders (20). The main stakeholders identified in the project included (i) participants of the intervention, (ii) care providers screening and referring people to the intervention, (iii) voluntary and community sector organizations that provide the necessary resources (e.g., activities, services, programs, expertise, connections), (iv) volunteers, and (v) the Spanish national healthcare and public health system supporting the adoption and implementation of NBSP (15).

Other stakeholders, such as facilitators organizing the activities or participants’ relatives, that may have also benefited from a broader perspective, are not included in this analysis.

### 2.2 Mapping inputs, outputs, and outcomes

Through initial engagement with stakeholders, a separate logic model of the overall RECETAS project showing the relationship between inputs, activities, and outcomes of the RECETAS trial was developed at the intervention planning stage (15). In alignment with this logic model, we created an impact map to illustrate the relationship between stakeholders inputs, outputs, and outcomes specifically for the FiN intervention in Barcelona (see Table 1). The participants, care providers, and volunteers input is limited to their time. The costs of intervention were covered through funding linked to the RECETAS project. Costs of the FiN intervention and for UC were collected in the trial in Barcelona. Total intervention costs (e.g., facilitators transport costs, personnel expenses, material costs) were calculated for UC and separately for the 15 FiN groups, each with seven to 14 participants, and divided by the number of participants to get the weighted average costs per participant. FiN intervention costs were supplemented by the session-specific costs of the 15 groups that arose to each participant during the nine sessions (e.g., public or private transportation, food and beverages). Specific costs in the UC group were collected from all participants (e.g., transportation costs, admission or participation in an activity, equipment or material costs) and considered at an individual level.

**Table 1:** Impact map – part 1.

Stakeholder	Input	Output
Participants of the intervention	Time	FiN: Group activity in nature including transport etc. UC: Signposting as usual care
Care providers	Time for screening and referring the participants to the intervention	
Voluntary and community sector organizations	Providing the necessary resources (activities, services, programs, expertise, connections, referral etc.)	
Volunteers	Time	
National healthcare and public health system	Supporting the adoption and implementation of a NBSP intervention	

FiN, Friends in Nature; NBSP, nature-based social prescribing; UC, usual care.

The output of the FiN intervention consisted of an individual interview followed by nine weekly group sessions over 3 months. Participants, together with the group facilitators, selected their preferred nature-based activities from a co-created menu developed alongside community stakeholders (22) or proposed activities they were aware of or wished to explore or even guided themselves. A diverse range of nature-based activities were performed: picnics in parks, group cohesion dynamics in green or blue spaces, walks through natural environments, guided visits to natural and heritage sites. Additional activities encompassed birdwatching, nature observation, forest bathing, mindfulness practices in nature, and artistic endeavors in outdoor settings, among others (23).

Participants assigned to the UC group also received an individual interview conducted by one of the two facilitators designated for each area. During this interview, they were informed of their group allocation, provided with the same co-created menu with nature-based activities they could join in their neighborhood, and encouraged to participate in selected activities or enhance their engagement with nature. A field diary was distributed for them to document during 9 weeks the duration of their contact with nature and the types of activities they engaged in (9).

### 2.3 Measuring, evidencing, and valuing outcomes

The following outcomes were identified as material from the impact map (see Table 2): The primary outcomes for the participants of the RECETAS project were reduced loneliness and improved health-related quality of life (HRQoL). Capability linked to the promotion of personal wellbeing (‘capability wellbeing’) was assessed as an alternative outcome to HRQoL as part of the sensitivity analysis. These three outcomes were measured at baseline and at 3 months follow-up. For loneliness, the De Jong-Gierveld Loneliness scale (DJGLS) (24) was used. The DJGLS ranges from 0 to 11 with a score of 2 or above indicating loneliness and 8 or above indicating strong loneliness (25). HRQoL was assessed with the EuroQol’s 5-level questionnaire (EQ-5D-5L) which was converted into a health utility score ranging from 0 (equivalent to death) to 1 (perfect health) using the Spanish value set (26, 27). Similarly, the ICEpop CAPability measure for Adults (ICECAP-A), which was used to assess capability wellbeing, was converted into a capability score ranging from 0 (no capability) to 1 (full capability) (28, 29).

**Table 2:** Impact map – part 2.

Stakeholder	Outcome	Outcome measure	Description of material change	Proxy (social value bank)	Monetary valuation source
Participants of the NBSP intervention	Loneliness	Loneliness score (DJGLS)	Improved loneliness by reduction ≥2 on the DJGLS scale	Being a member of a social group	Trotter, Vine (35)
	Health-related quality of Life	Health utility index (EQ-5D-5L)	Health utility score improvement of ≥0.1	Good overall health	Trotter, Vine (35)
	Capability wellbeing*	Capability index (ICECAP-A)	Capability score improvement of ≥ 0.1	Good overall health	Trotter, Vine (35)
Care providers	Reduced burden on the care system	This outcome is already considered in healthcare system	–	–	–
Voluntary and community sector organizations	Benefit from utilization of provided resources	Number of people reporting to take part in other group/social activities or clubs (Health economic questionnaire)	Difference between baseline and after 3-month intervention	Feel belonging to neighborhood	Trotter, Vine (35)
Volunteers	Uplift in life satisfaction caused by volunteering	Number of involved volunteers	Volunteering (yes/no)	Regular volunteering	Trotter, Vine (35)
National healthcare and public health system	Reduced burden on the health care system reduces	Health economic questionnaire	Difference of number of health resource use between baseline and after 3-months	Unit costs for health resource use (e.g., nurse, specialist)	Departament de Salut (Department of Health) (39)

*Only for sensitivity analysis; DJGLS, De Jong-Gierveld Loneliness scale; EQ-5D-5L, 5-level EQ-5D version; ICECAP-A, ICEpop CAPability measure for Adults.

Outcomes of NBSP also extend to care providers, as improved population health could reduce the burden on the care system by reducing patient visits to emergency services, inpatient and outpatient services, reduces provider fatigue, improves quality of interactions between provider and patients, and increases opportunities for the care providers to spend their time differently (e.g., focus on other patients, leisure time) (15). Voluntary and community sector organizations also benefit, as the implementation of NBSP programs could increase their reach to at-risk populations and the utilization of resources (activities, services, programs, expertise, connections, etc.). Similarly, volunteers could undergo an uplift in life satisfaction caused by volunteering (30, 31). Lastly, there are also benefits for the national healthcare and public health system, which experiences relevant changes or adaptations in the use of resources and reductions in use of prescribed medications (32, 33).

Outcome measures provide information about a material change of the stakeholders’ outcomes in order to give them a financial valuation. A change in the identified outcomes “was defined as material (i) when it was relevant to and consistent with the scope of the study and (ii) when it was significant in that it could influence decisions and inform good practice” (34).

For valuation of material changes, proxies have to be assigned to provide a monetary approximation to the outcomes that do not have a clear market price. Financial proxies were retrieved from the Social Value Bank (SVB) (35). The SVB provides a set of standardized monetary values for several social outcomes and reports values in British Pounds (GBP) per year per person based on wellbeing valuation (35). As, to our knowledge, no Spanish-specific social value proxies are currently available, UK-based values were used.

The value of GBP 1,850 for “being a member of a social group” (35) was used as an indicator for a reduction in loneliness if a material change in loneliness occurs through a reduction of two or more points on the DJGLS scale. The value of GBP 20,141 for “good overall health” was used as a proxy for improved HRQoL and capability wellbeing indicated by an improvement of ≥0.1 of the health utility score or capability score, respectively, (35, 36). As voluntary and community sector organizations benefit from the utilization of their provided resources, the number of people reporting participation in other group/ social activities or clubs was retrieved from the health economic questionnaire using baseline and 3-month follow-up data. The financial proxy of GBP 3,753 for “Feel belonging to neighborhood” was used (35). The number of volunteers involved was retrieved from the study site. A financial proxy for “Regular volunteering” of GBP 2,357 was defined for each volunteer (35).

All values from the SVB were converted to Euros and standardized to a common costing year using the Consumer Price Index (CPI) and the Purchasing Power Parity (PPP) (21). UK inflation using the CPI from 2014 to 2024 was considered first (37). After adjusting for inflation, the 2024 PPP rate between the UK and Spain was used to convert the adjusted GBP to Euros (38). Appendix Table A1 shows an exemplary calculation of how the CPI and PPP were used to adjust the social value for “being a member of a social group” of GBP 1,850 to 2024 Euros of 2,087 (35, 37, 38).

To estimate the outcome for the national healthcare and public health system, the difference of healthcare resources between baseline and after the three-month intervention (material per se) was estimated, measured as self-reported information from the health economic questionnaire in the trial. We assessed the visits to a primary care center or hospital, family doctor, specialist, nurse, psychologist, psychiatrist, emergency center, intensive care unit, rehabilitation services or physiotherapist, occupational therapist, social worker, telephone calls with the family doctor, and home visits by the family doctor, nurse, physiotherapist, occupational therapist, and social worker and valued the difference using Catalan public prices for health services (39). Unit prices were converted to 2024 Euros using the Spanish CPI (40). We assumed that outcome for care providers (i.e., reduced burden) was already considered in the outcome of the healthcare system.

### 2.4 Adjusting valued outcomes for real impact

In SROI analyses, valued outcomes are adjusted for deadweight, attribution, displacement, and drop-off. These four elements help to assess the true effect of an intervention and should be corrected for when calculating the social benefit of a program (41). Deadweight, attribution, displacement, and drop-off are measured as a percentage and are subtracted from the total outcome to estimate the net social benefit. Deadweight refers to the amount of outcome that would occur anyway and is not due to the intervention. Attribution describes the percentage of the outcome attributable to the intervention and not caused by other interventions or programs. Deadweight and attribution are typically applied in SROI studies as control groups are often not included, requiring adjustments to account for outcomes that would have occurred anyway and for effects attributable to other factors. As our study is based on an RCT with both an intervention and a control group, deadweight and attribution are inherently accounted for through the comparison between groups (21, 42, 43). Given the RCT design and the comparison between the treatment and UC groups based on differences in outcomes and costs, it was not necessary to apply deadweight and attribution adjustment factors. Displacement represents the extent to which the impact achieved displaces another possible outcome. As participants took part in the intervention voluntarily, we assumed that no alternative societal relevant activities, which could generate an outcome, were missed in the base-case analysis. Therefore, an adjustment for displacement was not considered necessary in the base-case analysis. However, the potential impact of displacement was examined in a scenario analysis. Drop-off as a correction factor means the reduction in outcome over time (20). Correcting for drop-off was not necessary as we were evaluating the SROI only over the time horizon of the trial.

### 2.5 Calculating the total social value, total costs, SROI ratio and NPV

To get the total social value, the value of each outcome was multiplied by the number of stakeholders experiencing each outcome. The SROI analysis included only participants with available three-month data on loneliness, HRQoL, or capability. A complete case analysis including only participants with complete data on loneliness, HRQoL, and capability was not considered due to missing outcomes. Further, no imputation was performed to address missing data. For health resource use referring to the national healthcare and public health system as a stakeholder, we used data from participants where at least one item from the health economic questionnaire was available. If another item was missing, we assumed there was no usage. Total costs were limited to the costs of the intervention in the treatment and UC groups.

Discounting the outcomes and costs over the duration of the intervention to get the present social value of each outcome and costs is recommended but since the intervention lasts less than a year, no discounting was necessary (44).

The difference of the total social value between FiN and UC per participant was divided by the difference in costs between FiN and UC per participant to calculate the SROI ratio expressing how much social value in Euro was generated per Euro invested in FiN:

$$\text{SROI ratio}=\frac{\begin{array}{l} \text{total social value}\;\mathrm{per}\;\mathrm{FiN}\;\text{participant}\\ -\text{total social value}\;\mathrm{per}\;\mathrm{UC}\;\text{participant} \end{array}}{\begin{array}{l} \text{total costs}\;\mathrm{per}\;\mathrm{FiN}\;\text{participant}\\ -\text{total costs}\;\mathrm{per}\;\mathrm{UC}\;\text{participant}\; \end{array}}$$

A SROI ratio above one means that a social value was generated by an investment in the intervention (21).

The NPV was calculated by the difference between the total social value and the total costs for FiN and UC participants:

$$\begin{array}{l} \mathrm{NP}V_{\mathrm{FiN}}=\text{total social value}\;\mathrm{per}\;\mathrm{FiN}\;\text{participant}\\ -\text{total costs}\;\text{value}\;\mathrm{per}\;\mathrm{FiN}\;\text{participant} \end{array}$$

$$\begin{array}{l} \mathrm{NP}V_{\mathrm{UC}}=\text{total social value}\;\mathrm{per}\;\mathrm{UC}\;\text{participant}\\ -\text{total costs}\;\mathrm{per}\;\mathrm{UC}\;\text{participant} \end{array}$$

A NPV above zero means that the intervention created more social value than it costs (45).

### 2.6 Sensitivity and scenario analyses

To test how sensitive our results are to different assumptions, we performed several sensitivity and scenario analyses for adjustment variables, financial valuations, outcome quantity, and inputs/costs (20).

Sensitivity Analysis 1: We calculated the SROI ratio and NPVs for the participants’ outcomes loneliness and capability wellbeing instead of HRQoL. This was done as capability wellbeing and HRQoL are similar concepts and to avoid overestimation of the total social value generated.

Sensitivity Analysis 2: We excluded research costs and session-specific costs from the intervention costs. Only trial costs were considered.

Sensitivity Analysis 3: We varied the social values from the SVB by ±10% to test the robustness of our assumption of applying UK values in the Spanish study setting (see Appendix Table A2).

Scenario Analysis 1: We defined a material change in loneliness if moving from “lonely” (any intensity) to “not lonely” according to the DJGLS scale and improved HRQoL indicated by an improvement of 20% or more of the health utility instead of a reduction of two points or more on the DJGLS scale and an improvement of ≥0.1 in HRQoL as applied in the main analysis.

Scenario Analysis 2: We applied a 5% displacement factor to the outcomes of participants of the FiN intervention to assess how the results would change if a proportion of participants’ outcomes would displace an outcome in another setting as a consequence of the intervention.

### 2.7 Validation

A quality assessment was performed, and results were validated with qualitative data.

#### 2.7.1 Quality assessment checklist

We compared two existing quality assessments checklists (43, 46). We decided to apply the 21-item quality assessment framework of Hutchinson et al. (46) as it is more comprehensive and captures almost all items from Krlev et al. (43). Only two items [8. Social effects captured? (qualitatively) & 9. Social effects captured? (quantitatively)] were not explicitly mentioned, which were additionally assessed (43).

#### 2.7.2 Qualitative data

Qualitative data from the RCT were used to validate our quantitative results. While stakeholders had supported the development of the logic model prior to the delivery of the intervention, as an ex-post assessment we sought to understand how the experiences of participants corresponded to the anticipated outcomes set out in the model and associated impact map (Table 2). This component of the study was intended to strengthen the SROI process through directly engaging our lead key stakeholder group—the intervention participants—on material changes and, in doing so, to support the validation of the SROI findings.

The qualitative data collection with participants took place at the end of the intervention with 10 focus groups (out of the 15 intervention groups; average five participants, minimum two, maximum 10), plus 12 individual interviews. Participants were asked open-ended questions concerned about their perceptions and experiences of the consequences (positive and negative) of taking part in the intervention. These data were supplemented with the records of 15 facilitator learning diaries (one for each group in the trial). The qualitative data were transcribed and thematically analyzed through deductive and inductive coding. Leading themes were defined as those where there were at least 10 separate illustrative reports in the dataset.

## 3 Results

### 3.1 Participant characteristics

The demographic characteristics of participants are presented in Table 3. Only participants with reported outcomes after 3 months were considered in the following SROI analysis. The number of participants who reported outcomes was slightly different for each variable studied loneliness, HRQoL, capability, and health resource use due to missing outcomes (see Table 3 and Appendix Figure A1).

**Table 3:** Demographic characteristics of participants.

Characterstics	FiN (*n* = 138)	UC (*n* = 116)
	Mean (SD)	Mean (SD)
Age	62.22	60.53
Gender, female	107	99
Gender, male	31	17
Loneliness (DJGLS)	*n* = 130	*n* = 103
Baseline	6.55 (3.11)	7.28 (3.07)
3 months	5.98 (3.28)	6.33 (3.44)
HRQoL (EQ-5D-5L)	*n* = 126	*n* = 102
Baseline	0.73 (0.18)	0.67 (0.24)
3 months	0.76 (0.20)	0.71 (0.23)
Capability (ICECAP-A)	*n* = 127	*n* = 100
Baseline	0.47 (0.24)	0.54 (0.24)
3 months	0.46 (0.21)	0.51 (0.23)
Health resource use (health economic questionnaire)	*n* = 126	*n* = 99

DJGLS, De Jong-Gierveld Loneliness scale; EQ-5D-5L, 5-level EQ-5D version; FiN, Friends in Nature; HRQoL, health-related quality of life; ICECAP-A, ICEpop CAPability measure for Adults; SD, Standard deviation; UC, usual care.

### 3.2 Valuing outcomes

The social and healthcare values converted to 2024 Euros are shown in Table 4. Additionally, outcomes for each stakeholder, the description of material change in each outcome, the respective financial proxy and the CPI and PPP-adjusted value are presented in Table 4.

**Table 4:** Impact map – part 3.

Stakeholder	Description of material change	Proxy	Value in 2024	Monetary valuation source (recalculated to EUR 2024)
Participants of the intervention	Improved loneliness by reduction ≥2 on the DJGLS scale	Being a member of a social group	EUR 2,087	Trotter, Vine (35)
	Health utility score improvement of ≥0.1	Good overall health	EUR 22,721	Trotter, Vine (35)
	Capability score improvement of ≥0.1*	Good overall health	EUR 22,721*	Trotter, Vine (35)
Care providers	–	–	–	–
Voluntary and community sector organizations	Difference between baseline and after 3-month intervention	Feel belonging to neighborhood	EUR 4,234	Trotter, Vine (35)
Volunteers	Volunteering (yes/no)	Regular volunteering	EUR 2,659	Trotter, Vine (35)
National healthcare and public health system	Primary care center or hospital visits	Non-urgent medical care health center	EUR 59.98	Departament de Salut (Department of Health) (39)
	Family doctor			
	Specialist	Urgent medical care health center	EUR 89.97	
	Nurse	Non-urgent nursing care health center	EUR 41.99	
	Psychologist	Mental health day hospital	EUR 147.98	
	Psychiatrist			
	Emergency center	Primary care emergency center	EUR 125.96	
	Intensive care unit	Intensive care stay	EUR 1,859.48	
	Rehab or physiotherapist	Socio-health day hospital	EUR 55.96	
	Occupational Therapist	Socio-health day hospital	EUR 55.96	
	Social worker	Socio-health day hospital	EUR 55.96	
	Phone call with family doctor	Telephone patient consultation	EUR 57.58	
	Home visits - family doctor	Non-urgent home medical care	EUR 95.97	
	Home visits - nurse	Non-urgent home nursing care	EUR 59.98	

*Only for sensitivity analyses.

### 3.3 Value of change

One hundred eight participants in the FiN group showed a material change in loneliness, and 38 in HRQoL. In the UC group, 82 participants showed a reduction in loneliness and 31 improved HRQoL.

Total healthcare resource use between baseline and the three-month follow-up was EUR 6,325 lower in the FiN and EUR 8,796 lower in the UC group (see Appendix Table A3). Table 5 presents the social value created for each stakeholder, the total impact, and the impact per participant with the FiN intervention compared to UC. No significant volunteering was identified in the trial, so that no social value was generated for volunteers in both groups. A total social value of EUR 1,145,897 for the FiN and EUR 892,727 for the UC group was generated (see Table 5). FiN generated a higher social value of EUR 276 per participant.

**Table 5:** Impact map – part 4.

Stakeholder	Outcome	Outcome measure	Quantity	Value	Impact	Impact per participant
Friends in nature						
Participants	Loneliness reduced	Improved loneliness by reduction ≥2 on the DJGLS scale	108/130	EUR 2,087	EUR 225,389	EUR 1,734
	Health-related quality of Life increased	Health utility score improvement of ≥0.1	38/126	EUR 22,721	EUR 863,379	EUR 6,852
	Capability wellbeing increased*	Capability score improvement of ≥0.1	36/127*	EUR 22,721*	EUR 817,938*	EUR 6,440*
Voluntary and community sector organizations	Benefit from utilization of provided resources	Difference between baseline and after 3-month intervention	12/138	EUR 4,234	EUR 50,804	EUR 368
Volunteers	Life satisfaction caused by volunteering	Volunteering (yes/no)	0	EUR 2,659	EUR 0	EUR 0
National healthcare and public health system	Reduced burden on the health care system reduced	Difference of number of health resource use between baseline and after 3-months	see Appendix Table A3		EUR 6,325 (savings)	EUR 50 (savings)
Total					EUR 1,145,897	EUR 9,004
Usual care						
Participants	Loneliness reduced	Improved loneliness by reduction ≥2 on the DJGLS scale	82/103	EUR 2,087	EUR 171,129	EUR 1,661
	Health-related quality of Life increased	Health utility score improvement of ≥0.1	31/102	EUR 22,721	EUR 704,336	EUR 6,905
	Capability wellbeing increased*	Capability score improvement of ≥0.1	19/100*	EUR 22,721*	EUR 431,690*	EUR 4,317*
Voluntary and community sector organizations	Benefit from utilization of provided resources	Difference between baseline and after 3-month intervention	2/116	EUR 4,234	EUR 8,467	EUR 73
Volunteers	Life satisfaction caused by volunteering	Volunteering (yes/no)	0	EUR 2,659	EUR 0	EUR 0
National healthcare and public health system	Reduced burden on the health care system reduced	Difference of number of health resource use between baseline and after 3-months	see Appendix Table A3		EUR 8,796 (savings)	EUR 89 (savings)
Total					EUR 892,727	EUR 8,729
Difference between the groups					EUR 253,169	EUR 276

*Only for sensitivity analysis.

### 3.4 Valuing inputs

Over all sessions, the intervention costs for FiN were accumulated to EUR 63,435 and to EUR 33,429 Euro for UC (see Table 6). The average costs, weighted by number of participants in each group, of the FiN intervention were EUR 369 per participant and EUR 208 per participant in the UC group. Separating total costs into research and trial costs, showed that the expenses for facilitators in the trial represented the main cost component in the FiN group. Adding intervention-specific costs that arose per group per session, total costs were EUR 67,034 for FiN and EUR 37,506 for UC, resulting in weighted average costs of EUR 419 per FiN and EUR 233 per UC participant. FiN cost EUR 186 more per participant than UC.

**Table 6:** Costs in 2024 Euro for FiN and UC.

Cost type	FiN (*n* = 160)	FiN per participant	UC (*n* = 160)	UC per participant	Difference between the groups
Research costs	27,079		26,830		
Trial costs	36,357		6,598		
Intervention costs	63,435	369	33,429	208	161
Intervention-specific costs	3,599		4,077		
Total costs	67,034	419	37,506	233	186

FiN, Friends in Nature; UC, usual care.

### 3.5 Calculating the SROI ratio and the NPV

In the base case, the FiN intervention generated a social return of EUR 1.48 per EUR 1 invested (see Table 7). The NPV was EUR 8,585 for each FiN and EUR 8,496 for each UC participant.

**Table 7:** Social return on investment ratios and net present value per participant in FiN and UC.

Per participant	FiN	UC	Difference
Total Social Value in EUR	9,004	8,729	276
Total Costs in EUR	419	233	186
SROI ratio	EUR 1.48:EUR 1		
NPV in EUR	8,585	8,496	90

FiN, Friends in Nature; NPV, net present value; SROI, social return on investment; UC, usual care.

### 3.6 Results of sensitivity and scenario analysis

Results of the sensitivity and scenario analyses are shown in Table 8. The SROI ratio increased when using capability wellbeing as an outcome measure instead of HRQoL, although the total social value and hence the NPV decreased. Excluding research costs and session specific costs led to a higher SROI ratio of EUR 1.71 per Euro invested and a higher NPV in both groups. Varying all social values that were retrieved from the SVB by +10% increased the total social value relatively more in FiN, so that the SROI ratio increased to EUR 1.65 per Euro invested. Lowering the social values by 10%, resulted in a smaller SROI ratio of EUR 1.31 per Euro invested. In scenario analysis 1, 22 participants improved in loneliness and 31 participants in HRQoL in FiN compared to 17 and 22 participants in UC. This smaller number of participants experiencing a material change led to a lower total social value and thus to a lower NPV. When applying a displacement rate of 5%, the FiN intervention generated less social value than UC and led to an SROI ratio of EUR 0.83 per Euro invested. The point of indifference for displacement was 3.2%, meaning that the FiN intervention would be favored if displacement remained below this threshold.

**Table 8:** Result of the sensitivity and scenario analyses.

Per participant	Sensitivity analysis 1 (ICECAP-A)			Sensitivity analysis 2 (only trial costs)			Sensitivity analysis 3 (social values ± 10%)			Scenario analysis 1 (material change scenario)			Scenario analysis 2 (displacement scenario)		
	FiN	UC	Difference	FiN	UC	Difference	FiN	UC	Difference	FiN	UC	Difference	FiN	UC	Difference
Total social value in EUR	8,593	6,140	2,452	9,004	8,729	276	9,900/8,109	9,593/7,865	307/244	6,361	5,407	955	8,575	8,729	−154
Total costs in EUR	419	233	186	369	208	161	419	233	186	419	233	186	419	233	186
SROI ratio	13.18:1			1.71:1			1.65:1/1.31:1			5.13:1			0.83:1		
NPV in EUR	8,174	5,907	2,266	8,635	8,521	115	9,481/7,690	9,360/7,632	121/58	5,942	5,174	769			

EUR, Euro; FiN, Friends in Nature; ICECAP-A, ICEpop CAPability measure for Adults; NPV, net present value; UC, usual care.

### 3.7 Validation results

#### 3.7.1 Quality assessment checklist

The quality assessment checklist can be found in Appendix Table A4. Overall, all 21 items of the primary checklist were fulfilled (46). The two additional assessed items evaluated were also fulfilled (43).

#### 3.7.2 Qualitative data results

An initial qualitative analysis of the participants’ focus groups and interviews, and facilitators’ learning diaries, was undertaken to identify perceived outcomes and thus help reinforce the results of the SROI. Participants reported that they experienced positive changes with respect to reductions in experiences and feelings of loneliness, and an improved sense of connectedness with others. This was also reflected in expressions of an improved sense of wellbeing and ability to better look after their personal health.

Leading themes from participants reflected a range of health and wider related benefits that included: addressing long term health issues (including mental health conditions); managing experiences of chronic inactivity or low energy; addressing life changes linked to, for example, bereavement or loss of a work role; providing a sense of belonging to a group; sharing experiences in common with others; regaining trust in other people; and confidence to engage in new hobbies and leisure activities.

Participants also reported how engagement with FiN helped them form or regain connections with their neighborhood, towns, or local surroundings. This was supported through greater confidence and knowledge of options to experience the natural environment (e.g., through new knowledge of public transport) and greater interest and value placed on the natural environment.

Less common themes reflect other effects of the intervention on a range of issues including suicidal ideation; relationship break-up; improved relationships in community workplace or other settings; and feeling of greater self-worth and validation in social group settings.

Feedback from participants also showed that there were some unanticipated negative outcomes. These included:

- Conflict between participants in the group due to differences in personality or social circumstances.
- Feelings of discomfort, distress or disconnection with others due to the anxieties in group situations and personal comparisons.
- Physical injury while taking part in a nature-based activity.

These types of situations led to the early withdrawal of participants from the intervention. However, all were unusual and only occasionally reported.

## 4 Discussion

The SROI ratio supports more informed decision-making by incorporating social value alongside financial considerations. We conducted a SROI analysis to quantify the broader benefits of investing in a NBSP intervention to reduce loneliness. We used empirical data from the RECETAS RCT in Barcelona, Spain, and compared the NBSP intervention FiN with UC. The SROI ratios remained consistently favorable across the base-case and the sensitivity analyses, ranging from 1.31 to 13.18. The NPV also remained positive and consistently favored the FiN intervention, indicating a higher social value than total costs in the base-case and the sensitivity analyses. When using the ICECAP-A measure of capability wellbeing instead of HRQoL as the outcome, the impact in the FiN group remained largely unchanged. However, the impact in the UC group decreased, resulting in a larger per-participant difference between groups, explaining the higher SROI ratio observed. Importantly, the analysis within the RECETAS trial included intervention development costs that extended beyond the direct trial costs. If in future, the Spanish national healthcare and public health system might act as the funding agency for the intervention, there would be no development costs. Sensitivity analysis showed that the SROI ratio was higher if these costs were excluded. SROI ratios remained above 1 when varying the applied social values by ±10%. Scenario analysis 1 indicated that the SROI ratio increased. In contrast, assuming a displacement factor of 3.2% to FiN resulted in indifference between FiN and UC. Our results are in line with previous studies evaluating the SROI of socially prescribed nature-based interventions for mental health problems, which showed positive SROI ratios ranging from 2.57 to 4.67 (11), respectively 3.30–4.70 per GBP invested (12).

The qualitative findings on perceived outcomes provided three areas of contribution toward the SROI process. Firstly, through corroborating the importance of the key outcomes (loneliness, health-related quality of life, capability) the results helped validate the SROI results. Secondly, analysis of the reports from participants indicated that there was a wide range of outcomes that were not identified in the initial logic model and impact mapping process. These supplementary outcomes point toward additional forms of value created through the intervention. Finally, the qualitative findings provide a basis for further development of the RECETAS intervention to enhance specific health-related benefits, social activities and connectedness to the community and natural environment. Potential negative outcomes identified indicate the importance of managing group dynamics, personalized support and risk management in the process of delivering the intervention.

In future work, the analysis could be extended to include additional trial sites within the RECETAS project to enhance generalizability and external validity. Future research could also explore the social and emotional dimensions of loneliness separately to better identify which aspects are most responsive to NBSP interventions. Moreover, further longitudinal evidence would strengthen understanding of post-intervention effects; for instance, improvements in capability wellbeing may translate into changes in healthcare utilization and community engagement (such as volunteering) over time.

### 4.1 Strengths

Previous studies have investigated the impact of social prescribing by calculating its SROI ratio (11, 12, 47). However, this is the first SROI study evaluating a NBSP intervention against loneliness following the six steps of the SROI guide (20). A key strength of this study is that the SROI study was based on quantitative empirical data from a previously conducted RCT. We used the financial proxies from the SVB to value outcomes based on the wellbeing valuation method which we converted to the current price year. Comparing the FiN group with UC reduced the risk of overestimating the SROI of the intervention effect. We also performed a sensitivity analysis for the outcome loneliness and capability wellbeing to examine the effects of different outcome measures. In addition to the SROI ratio, we estimated the NPV for both groups as a second summary measure.

Unlike cost-effectiveness analyses, the concept of SROI captures a broader range of social, wellbeing, community, and environmental benefits that are often highly relevant to participants and stakeholders. By monetizing these diverse outcomes, results of the SROI study enable to reflect impacts without a market value, such as improvements in social connectedness, empowerment, and community cohesion, making it especially suitable for evaluating interventions with wide-ranging societal effects.

In addition, we used qualitative data from trial participants to validate our results and performed a quality assessment with two checklists.

### 4.2 Limitations

This study has several limitations. Only primary outcomes were considered in the main SROI analysis. Secondary outcomes related to psychosocial health as identified in the logic model could have further impact on the social value generated (15). Another limitation is that not all outcomes were available in the SVB, so that a proxy was needed and thus may not be optimal. For instance, the proxy value for “Feel belonging to neighborhood” (GBP 3,753 per person per year) could alternatively be applied to the loneliness outcome (35). In addition, no Spanish social value sets were available, so that GBP prices had to be converted to Spanish Euros. The GBP values come from UK studies based on wellbeing valuation. Cultural comparability and valuation stability may not be given and social values might be different in Spain. For example, subjective wellbeing is slightly lower than in the UK (48) and the transferred social values could be either higher or lower in Spain and may not fully reflect local preferences or valuations. Further stakeholder engagement and considering more outcomes as well as other proxy values, might also lead to a different SROI ratio.

The DJGLS distinguishes between social and emotional dimensions of loneliness. In this study, we focused on the total loneliness score without differentiating between these subdimensions. It is possible that the nature and group components have addressed both subtypes but have not analyzed the specific impacts at these levels.

Sample sizes for each outcome are different. However, we used as much outcome data as possible from the trial site. Due to drop-outs and missing cases at three-month follow-up, particularly in the control group, we have not been able to analyze all initially randomized participants limiting validity of our results. Also, no imputation of missing data was performed. Our assumption that no answer in a resource use item means no usage could lead to over- or underestimation in health resource use. However, as it affects both groups the effect might be negligible. Moreover, at this stage of the study, self-reported data were available but not data through medical records, which would be more reliable.

FiN is a holistic group-based model of NBSP with elements adapted from “Circles of Friends” reinforcing the psychosocial and group dynamics and thus adopts a different approach than other models of NBSP (49). Therefore, these results may not be generalizable to other models of NBSP without the specific FiN characteristics. Additionally, the intervention’s three-month time frame represents a limitation, as it remains unclear whether the observed effects are temporary or persistent or change over time.

In addition, the methodology of this SROI study was reported in the initial RECETAS project proposal but not published in a separate protocol. In doing so, potential bias from the researchers could have been reduced.

## 5 Conclusion

Overall, the FiN intervention compared with UC generated a favorable SROI, with SROI ratios above one across most of our analyses. If FiN would displace potential outcomes in another sector, UC might be favored. This indicates that the intervention could generate social value relative to its costs, supporting its potential to reduce loneliness and improve wellbeing. Also, the FiN intervention’s present value exceeded its costs and stayed above the NPV of UC in most analyses. The findings were also consistent with previous studies evaluating the SROI of nature-based social prescribing for mental health. Qualitative findings further supported the analyses by validating key outcomes, revealing additional areas of value creation not captured in the initial theory of change, and highlighting opportunities to enhance health-related benefits, social activities, and community connectedness. Overall, these findings suggest that incorporating nature-based interventions into public health strategies could generate meaningful social and economic benefits. However, we emphasize that the findings are context-dependent and should be interpreted in light of the specific study setting, population characteristics, and implementation conditions, as transferability to other contexts and target groups may be limited.

## Data Availability

The datasets presented in this article are not readily available because the data analyzed in this study was obtained through a data transfer agreement between ISGlobal and UMIT TIROL. Requests to access the datasets should be directed to Jill S. Litt, jill.litt@isglobal.org.
