# Supplementary material for: Evaluating the social return on investment of nature-based social prescribing to alleviate loneliness: a randomized controlled trial in Barcelona
Source: Front Public Health. 2026 May 25;14:1774155. doi: 10.3389/fpubh.2026.1774155 (PMC13244874; doi:10.3389/fpubh.2026.1774155)
Supplement: Supplementary file 1 [file Supplementary_file_1.docx]

## Appendix

| *Table A1. CPI and PPP Adjustments – Calculation Example for Outcome* *Loneliness* | | | | | | | | |
| --- | --- | --- | --- | --- | --- | --- | --- | --- |
| **Outcome** | **Outcome measure** | **Description of material change** | **Proxy** | **Unadjusted value in GBP** | **GBP CPI: 2014 - 2024** | **Value in 2024 GBP – CPI adjusted** | **PPP 2024:**  **UK – Spain** | **Value in 2024 EUR – PPP adjusted for Spain** |
| Loneliness | Loneliness score (DJGLS) | Improved loneliness by reduction ≥2 on the DJGLS scale | Being a member of a social group | GBP 1,850  retrieved from Trotter, Vine (1) | 1.34  retrieved from ONS (2) | GBP 1,850 * 1.34 =  GBP 2,477 | 0.84 retrieved from OECD (3) | GBP 2,477 * 0.84 =  EUR 2,087 |
| Note: CPI, Consumer Price Index; DJGLS, De Jong-Gierveld Loneliness scale; PPP, Purchasing Power Parity; OECD, Organisation for Economic Co-operation and Development; ONS, UK Office for National Statistics. | | | | | | | | |


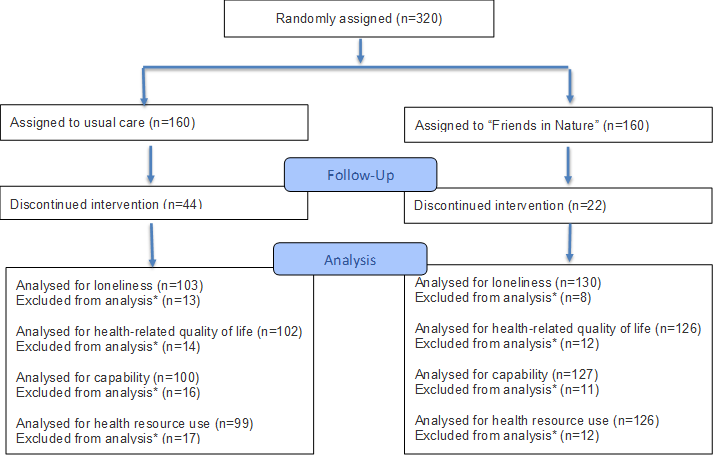


*Figure A1: Flow Diagram adapted from CONSORT 2025 (4)*

* Participants did not complete respective outcome measure at follow-up.

Note. Loneliness was measured with De Jong-Gierveld Loneliness scale, health-related quality of life with the EQ-5D-5L, and capability with the ICEpop CAPability measure for Adults.

| *Table A2. Social Values used in Sensitivity Analysis 3 – Varying the Social Values from the Social Value Bank (1) by ±10%* | | | |  |
| --- | --- | --- | --- | --- |
| **Proxy** | **Value in 2024 EUR** | **Value in 2024 EUR**  **+ 10 %** | **Value in 2024 EUR**  **- 10 %** | |
| Being a member of a social group | 2,087 | 2,296 | 1,878 | |
| Good overall health | 22,721 | 24,993 | 20,448 | |
| Feel belonging to neighborhood | 4,234 | 4,657 | 3,810 | |
| Regular volunteering | 2,659 | 2,925 | 2,393 | |

| *Table A3. Healthcare resource use at baseline and after three months.* | | | | | | | | |
| --- | --- | --- | --- | --- | --- | --- | --- | --- |
| **National healthcare and public health system** | | | | | | | | |
| **Have you visited a primary care center or hospital?** | **Group (cases with health resource data at 3 months n=126 for FiN, n=99 for UC)** | **Baseline** | **3 months** | **Difference in frequency** | **Cost per visit (EUR 2024)** | **Total cost difference** | **Average cost difference per participant** | **Monetary Valuation Source** |
|  |  |  |  |  |  |  |  |  |
|  |  |  |  |  |  |  |  |  |
| Primary care center or hospital | FiN | 113 | 114 | 1 | 59.98 | 59.98 | 0.48 | Departament de Salut (Department of Health) (5).  Recalculated to Euro 2024. |
| Primary care center or hospital | UC | 86 | 90 | 4 |  | 239.93 | 2.42 |  |
| **Frequency of visits to:** | | | | | | | |  |
| Family Doctor | FiN | 199 | 187 | -12 | 59.98 | -719.80 | -5.71 |  |
| Family Doctor | UC | 169 | 122 | -47 |  | -2,819.21 | -28.48 |  |
| Specialist | FiN | 156 | 138 | -18 | 89.97 | -1,619.54 | -12.85 |  |
| Specialist | UC | 162 | 157 | -5 |  | -449.87 | -4.54 |  |
| Nurse | FiN | 137 | 137 | 0 | 41.99 | 0.00 | 0.00 |  |
| Nurse | UC | 123 | 168 | 45 |  | 1,889.47 | 19.09 |  |
| Psychologist | FiN | 101 | 112 | 11 | 147.98 | 1,627.76 | 12.92 |  |
| Psychologist | UC | 87 | 86 | -1 |  | -147.98 | -1.49 |  |
| Psychiatrist | FiN | 29 | 31 | 2 | 147.98 | 295.96 | 2.35 |  |
| Psychiatrist | UC | 28 | 27 | -1 |  | -147.98 | -1.49 |  |
| Emergency Center | FiN | 52 | 50 | -2 | 125.96 | -251.93 | -2.00 |  |
| Emergency Center | UC | 72 | 30 | -42 |  | -5,290.51 | -53.44 |  |
| Intensive Care Unit | FiN | 2 | 0 | -2 | 1859.48 | -3,718.95 | -29.52 |  |
| Intensive Care Unit | UC | 1 | 0 | -1 |  | -1,859.48 | -18.78 |  |
| Rehab or Physiotherapist | FiN | 107 | 75 | -32 | 55.96 | -1,790.86 | -14.21 |  |
| Rehab or Physiotherapist | UC | 102 | 126 | 24 |  | 1,343.14 | 13.57 |  |
| Occupational Therapist | FiN | 2 | 3 | 1 | 55.96 | 55.96 | 0.44 |  |
| Occupational Therapist | UC | 2 | 1 | -1 |  | -55.96 | -0.57 |  |
| Social Worker | FiN | 62 | 29 | -33 | 55.96 | -1,846.82 | -14.66 |  |
| Social Worker | UC | 50 | 40 | -10 |  | -559.64 | -5.65 |  |
| Phone call with family doctor | FiN | 22 | 32 | 10 | 57.58 | 575.84 | 4.57 |  |
| Phone call with family doctor | UC | 27 | 22 | -5 |  | -287.92 | -2.91 |  |
| Home visits - Family Doctor | FiN | 0 | 8 | 8 | 95.97 | 767.78 | 6.09 |  |
| Home visits - Family Doctor | UC | 2 | 4 | 2 |  | 191.95 | 1.94 |  |
| Home visits - Nurse | FiN | 0 | 5 | 5 | 59.98 | 299.92 | 2.38 |  |
| Home visits - Nurse | UC | 1 | 0 | -1 |  | -59.98 | -0.61 |  |
| Home visits - Physiotherapist | FiN | 0 | 0 | 0 |  | 0.00 | 0.00 |  |
| Home visits - Physiotherapist | UC | 0 | 0 | 0 |  | 0.00 | 0.00 |  |
| Home visits - Social Worker | FiN | 0 | 0 | 0 |  | 0.00 | 0.00 |  |
| Home visits - Social Worker | UC | 0 | 0 | 0 |  | 0.00 | 0.00 |  |
| Home visits - Occupational therapist | FiN | 0 | 0 | 0 |  | 0.00 | 0.00 |  |
| Home visits - Occupational therapist | UC | 0 | 0 | 0 |  | 0.00 | 0.00 |  |
| **TOTAL** | FiN |  |  |  |  | **-6,264.70** | **-49.72** |  |
| **TOTAL** | UC |  |  |  |  | **-8,014.04** | **-80.95** |  |
| **Difference between the groups** |  |  |  |  |  | **1,749.35** | **31.23** |  |

| *Table A4. Quality Assessment of the SROI study (Hutchinson et al., 2019; Krlev, G. et al., 2013)* | | |
| --- | --- | --- |
| **Hutchinson et al., 2019** |  |  |
| **Research Question** | **Scoring** | **Notes** |
| Was a well-defined question posed? | Yes, p. 6 | The research question evaluating the social return on investment of nature-based social prescribing (NBSP) alleviating loneliness was explained in the Introduction. |
| **Reason for use of SROI Method** |  |  |
| Were authors transparent about why SROI methodology was chosen? (e.g. strategic planning/funding requirements) | Yes, p. 6f. | SROI methodology was chosen as part of the health economic evaluations of the RECETAS project and was explained in Introduction and Methods. |
| Did authors report relevant background literature / justify the need for the study? | Yes, p. 5f. | Reported in the Introduction and Methods. |
| **Scope** |  |  |
| Was the range of stakeholders included/excluded justified? | Yes, p. 7 | Key stakeholders identified, reason for inclusion reasons and excluded stakeholders were described in the Methods. |
| Was the range of stakeholders wide enough to adequately answer the research question? (principle of understanding change) | Yes, p. 7 | Stakeholders selection justified in the Methods. |
| Was it clear how stakeholders were involved and what data would be gathered from them? | Yes, p. 12 | Stakeholders involvement was provided in the Methods section. |
| Was ethics obtained/informed consent provided? | Yes, p. 26 | Ethical approval was provided. |
| **Theory of change/impact map** |  |  |
| Was the theory of change clear? i.e. the relationships between inputs, outputs and outcomes | Yes, p. 7f. | An impact map was presented in the Methods section. A referral to the logic model was provided. |
| Were unintended outcomes (positive/negative) detailed? | Yes, p. 15 | Yes, unintended outcomes recorded in the trial site were reported in the qualitative findings. |
| **Study Design** |  |  |
| Was the study design appropriate for the study question? (Control group, pre-post) | Yes, p. 5f. | The RCT study design was explained in the Introduction and Methods. |
| Was the sample described in detail/was the sample justified? | Yes, p. 6 | Participants eligibility criteria were explained in the Methods. |
| **Analysis** |  |  |
| Were inputs clear with non-monetized inputs valued appropriately? | Yes, p. 8f. | The valuation of inputs was explained in the Methods. |
| Were capital costs, as well as operating costs included? | Yes, p. 8 | The costs included were described in the Methods. |
| Were costs that occur in the future ‘discounted’ to their present values? Was justification given for the discount rate used? | Yes, p. 11 | The reason for not discounting was explained in the Methods. |
| Was dead-weight clearly described and calculated? | Yes, p. 10 | Deadweight was described in the Methods and presented in the Results. |
| Were the indicators valid and comprehensive? (Were the sources of all values clearly identified?) | Yes, p. 9f. | Indicators for the outcome and material change were described in the Methods. |
| Were the proxies valid and comprehensive? (Were the sources of all values clearly identified?) | Yes, p. 9f. | Financial proxies assigned from the UK Social Value Bank were described in the Methods. |
| Was length of benefit established and justified? (Drop-off) (In capital projects, did authors establish and differentiate between length of benefit and life expectancy of the asset?) | Yes, p. 10 | The length of benefit was three months which was the duration of the intervention. This is described in the Methods. |
| Were limitations and biases reported? | Yes, p. 17f. | Limitations were reported in the Discussion. |
| Was the final SROI ratio interpreted? | Yes, p. 15. | SROI ratios were interpreted in the Discussion. |
| Was sensitivity analysis performed? Was justification provided for the range of values (or for key study parameters) in the sensitivity analysis? | Yes, p. 11, 14 | Performed sensitivity analyses were described in the Methods and reported in the Results. |
| **Krlev, G. et al., 2013** |  |  |
| 8. Social effects captured? (qualitatively) | Yes, p. 14f. | Qualitative data from stakeholder involvement was incorporated. |
| 9. Social effects captured? (quantitatively) | Yes, p. 13f. | Quantitative effects were considered. |

**References**

1. Trotter L, Vine J, Leach M, Fujiwara D. Measuring the Social Impact of Community Investment: A Guide to using the Wellbeing Valuation Approach. HACT (Housing Associations' Charitable Trust); 2014.

2. Office for National Statistics (UK). (Release date: 18 September 2024). Consumer price inflation tables, table 20a. Retrieved 08.10.2024 from <https://www.ons.gov.uk/economy/inflationandpriceindices/datasets/consumerpriceinflation>

3. OECD. (2024). Purchasing power parities database. Retrieved 08.10.2024 from [https://data-explorer.oecd.org/vis?tm=PPP%20detailed%20&pg=0&snb=32&df[ds]=dsDisseminateFinalDMZ&df[id]=DSD_PPP%40DF_PPP&df[ag]=OECD.SDD.TPS&df[vs]=&pd=%2C2023&dq=.A.PPP...OECD&to[TIME_PERIOD]=false&vw=tb](https://data-explorer.oecd.org/vis?tm=PPP%20detailed%20&pg=0&snb=32&df%5bds%5d=dsDisseminateFinalDMZ&df%5bid%5d=DSD_PPP%40DF_PPP&df%5bag%5d=OECD.SDD.TPS&df%5bvs%5d=&pd=%2C2023&dq=.A.PPP...OECD&to%5bTIME_PERIOD%5d=false&vw=tb)

4. Hopewell S, Chan AW, Collins GS, Hrobjartsson A, Moher D, Schulz KF, Tunn R, Aggarwal R, Berkwits M, Berlin JA, Bhandari N, Butcher NJ, Campbell MK, Chidebe RCW, Elbourne D, Farmer A, Fergusson DA, Golub RM, Goodman SN, Hoffmann TC, Ioannidis JPA, Kahan BC, Knowles RL, Lamb SE, Lewis S, Loder E, Offringa M, Ravaud P, Richards DP, Rockhold FW, Schriger DL, Siegfried NL, Staniszewska S, Taylor RS, Thabane L, Torgerson D, Vohra S, White IR, Boutron I. CONSORT 2025 statement: updated guideline for reporting randomised trials. BMJ. 2025;389:e081123.

5. Departament de Salut (Department of Health). ORDRE SLT/63/2020, de 8 de març, per la qual s'aproven els preus públics del Servei Català de la Salut. Diari Oficial de la Generalitat de Catalunya 8134, 08/03/2020 (Order SLT/63/2020, of March 8, approving the public prices of the Catalan Health Service. Official Journal of the Generalitat of Catalonia 8134, 08/03/2020). 2020.
